# Moderating Role of Coping Style on the Relationship between Stress and Psychological Well-Being in Hong Kong Nursing Students

**DOI:** 10.3390/ijerph191811822

**Published:** 2022-09-19

**Authors:** Winnie Lai Sheung Cheng, Phyllis Man Chi Young, Kevin Kar Ho Luk

**Affiliations:** School of Nursing, Tung Wah College, Hong Kong, China

**Keywords:** stress, coping, psychological well-being, nursing students

## Abstract

Aims and objectives: To examine whether coping styles moderate the influence of stressors and psychological well-being in Hong Kong nursing students. Background: Stress could contribute to psychological distress in nursing students. Coping strategies are essential to mitigate psychological distress. So far, the moderating effects of coping between stressors and psychological well-being has not been thoroughly investigated. Design: This is a cross-sectional study conducted at four higher education institutions in Hong Kong. Methods: We recruited a convenience sample of 293 nursing students in February 2018. The Stressors in Nursing Students Scale-Chinese version (SINS-CN), Brief Cope Inventory-Chinese version (Brief COPE-C), and the Chinese version of the General Health Questionnaire-12 (C-GHQ-12) were used to measure the stressors, coping styles, and psychological well-being, respectively. Three multiple hierarchical linear regression models were used to identify the associations between the variables. Results: The stressors related to clinical learning, confidence, and personal problems were significant in explaining the psychological well-being. The coping strategies also predicted the psychological well-being and explained 44.5% of the variance. The coping strategy—accommodation—moderated the relationship between personal problems and psychological well-being. Conclusion: Problem-solving and accommodation types of coping were adaptive to stress and effective in promoting psychological well-being. However, using accommodation to cope with stressors related to personal problems will exacerbate the negative effects of the personal problems on the psychological well-being. Relevance to clinical practice: This study reveals the relationships between stressors, coping, and psychological well-being. Nurse educators must be aware of nursing student coping styles so they may devise strategies to promote effective coping to reduce the psychological distress among nursing students.

## 1. Introduction

Nursing is well-known to be a stressful occupation [1,2]. Nursing students share some of the sources of stress experienced by qualified nurses [3]. High levels of stress can affect their academic performance and result in dropping out from courses [4]. In addition, prolonged stress leads to psychological and physiological ill-health and increases the risk of mental disorder [1,2,5]. Concerns regarding the levels of stress and its effects on academic performance and psychological well-being in nursing students have led to recent attempts to explore the sources of stress, methods of coping, and the relationship between stress and psychological well-being in nursing students.

Sources of stress in nursing students mainly arise from clinical learning, academic study, and issues in their social lives [6,7,8]. The clinical learning-related stressors include a lack of knowledge and skills to perform the patient care, and heavy workloads [8,9,10,11]. The academia-related stressors include a fear of examinations and assignments [8,12]. The sources of stress related to their personal affairs include finance [13], negative relationships with the ward staff and/or faculty [7], and health problems [14]. Knowing how and where stress arises enables one to cope with it more effectively, and thus mitigate its detrimental effects. To deal with stress, coping mechanisms are necessary. Lazarus & Folkman’s [15] Transactional Model of Stress identifies two types of coping: problem-based and emotion-based. Coping is an adaptive process based on primary and secondary appraisals [15]. Ineffective coping with stress may present with signs of an inability to release tension and/or to control emotional responses, and of impaired functioning. The failure to cope heightens anxiety, leading to both physiological and psychological symptoms [16,17]. Several studies have found that problem-based coping has beneficial effects on nursing students’ academic performance and psychological well-being [7,17,18], whereas the nursing students who use emotion-based coping are prone to have negative health outcomes [19]. An international study revealed that the coping styles used by nursing students in each country are different [20]. In examining Chinese societies, one study found that nursing students in China were more likely to use an avoidance behavior when facing stress during clinical practice [21]. In Hong Kong, Chan et al. [10] reported that, though nursing students used transference frequently, they also used problem-solving strategies when encountering higher levels of stress related to patient care, and avoidance strategies when encountering higher levels of stress concerning interactions with the ward staff and faculty.

The previous studies have focused on identifying the sources of stress, measuring the levels of each, and investigating how nursing students cope with them. There is limited evidence as to the effects of different stressors and the efficacy of nursing students’ coping styles on their psychological well-being. In Hong Kong, a shortage of nurses has already been a problem. It is predicted that there will be a shortfall of nursing manpower of 1669 nurses in 2030 [22]. Given that fact, training new nurses to enter the workforce is critical. Stress and its related experience, such as burnout, has been cited as a reason that nursing students drop out of nursing programs [12,23,24]. Therefore, as a means to ensure adequate numbers of nurses in the future, it is imperative to explore the factors that give rise to stress and to determine the most effective ways of coping with it. This is the first study attempting to examine how different styles and strategies of coping with stress affect the psychological well-being of nursing students.

## 2. The Study

### Aim

This study aimed to explore the relationship between the sources of stress and psychological well-being in nursing students in Hong Kong.

This study addressed two research questions:

1. What is the relationship between the sources of stress and psychological well-being in nursing students?
2. What role does coping style play in moderating stress and psychological well-being?

## 3. Methods

### 3.1. Study Design and Settings

This study used a cross-sectional, descriptive design to collect data via a questionnaire-based survey. Based on the G*Power 3 software (version 3.1.9.7; Heinrich-Heine-Universität Düsseldorf, Düsseldorf, Germany), a sample size of >238 is required for multiple regression analysis to yield a power of 95%, with a medium effect size of 0.15, at a significance level of 0.05, and with 24 predictors [25]. A convenience sample of 293 nursing students from three higher education institutes consented to participate in the study (293/475; response rate = 61.7%). The participants were invited either during a mass lecture or through social media. These three institutes represented both government and privately funded nursing programs. The inclusion criteria were students in the final year of study in a baccalaureate nursing program. Final-year nursing students were selected because they had more academic and clinical experience to draw on. Nursing students studying mental health degree programs were excluded.

### 3.2. Ethical Considerations

This study was approved by the college research committee (NUR/SRC/20171220/036). The data were collected in February 2018. Before the data collection, the participants were informed about the study and their rights. Information about the study was given. Consent for the study was implied by the return of the completed anonymous survey.

### 3.3. Measures

#### 3.3.1. Stressors in Nursing Students Scale-Chinese Version (SINS-CN)

This is a 43-item scale originally developed by Deary et al. (2003) to examine the sources of stress. The SINS-CN was validated in a population of nursing students in Macao, a Special Administrative Region of the Republic of China [26]. It assesses five dimensions: clinical learning (17 items), academic study (4 items), finance and time (7 items), confidence (11 items), and personal problems (4 items). The factor loadings of the five dimensions ranged from 0.42 to 0.79; Cronbach’s alpha was 0.96 for the overall scale and ranged from 0.67 to 0.94 for the factors. The SINS-CN uses the five-point Likert type scale from 1 = ‘not at all stressful’ to 5 = ‘extremely stressful’. In this study, Cronbach’s alpha for the entire scale was 0.928.

#### 3.3.2. Brief Cope Inventory-Chinese Version (Brief COPE-C)

This 28-item scale was originally developed by Carver [27]. The Chinese version of the Brief COPE inventory based on 11 factors was adopted in this study [28]. The 28 items were categorized as problem-solving (4 items), accommodation (4 items), support-seeking (4 items), substance use (2 items), self-blame (2 item), venting (2 items), denial (2 items), behavioral disengagement (2 items), religion (2 items), self-distraction (2 items) and humor (2 items). The scale is rated with a four-point Likert scale, ranging from 1 (‘I haven’t been doing this at all’) to 4 (‘I’ve been doing this a lot’). A higher total score indicates more frequent use. The convergent validity of the 11 factors was supported by the correlation coefficients and stepwise multiple regressions. In this study, Cronbach’s alpha for the entire scale was 0.748.

#### 3.3.3. Chinese Version of the General Health Questionnaire-12 (C-GHQ-12)

This is a 12-item scale originally developed by Goldberg [29]. The C-GHQ-12 was used to measure the psychological distress [30]. The scale assesses to what extent the respondent has experienced a particular symptom or behavior in the past four weeks. The C-GHQ-12 has good internal consistency (Cronbach’s alpha, 0.87) [31]. The Likert scoring method was used, with each of the twelve items scored in the range 0 to 3 (0 = not at all, 1 = occasionally, 2 = sometimes, or 3 = always). In this study, Cronbach’s alpha for the entire scale was 0.689.

The remaining items recorded demographics including gender, age, studying institution, and religious belief.

### 3.4. Data Analysis

We analyzed the data using the IBM SPSS Statistics for Windows, software version 26 (IBM Corp, Armonk, NY, USA). The descriptive statistics of the frequencies, means and standard deviation were reported for all the variables. A Pearson Product-Moment correlation was used to investigate the relationships between the stressors, coping styles, and psychological well-being. A hierarchical multiple regression was conducted to establish the moderators and mediators. In model 1, we entered the sources of stress relating to the primary appraisal and the measures on gender, age, and religious belief. In model 2, we entered the secondary appraisal relating to the coping styles. For the final model, we entered the moderators that had been identified, after testing for the interaction effects between each predictor and the potential moderator variable. We conducted separate regressions to establish the moderators, according to the guidelines proposed by Baron and Kenny [32]. Those interaction variables that explained a statistically significant amount of variance in the psychological well-being were entered into the final model of the hierarchical multiple regression.

For all the statistical tests, the variables were considered significant at a significance level of 0.05.

## 4. Results

### 4.1. Demographics, Stress, Coping Style, and Psychological Well-Being of the Participants

A total of 293 nursing students participated in the study. The age of the students ranged from 22 to 30, with a mean age (SD) = 22.92 (1.07) years. The majority were female (female = 233; 79.5%, male = 60; 20.5%). Most reported to have no religious belief (n = 228; 77.8%). Two-thirds were from two privately funded institutions (A, n = 168, 57.3%; B, n = 37, 12.6%), and the remaining one-third were from a publicly funded institution (C, n = 88, 30.0%).

The mean score of the SINS-CN was 3.05 (SD = 0.44), indicating that the overall level of stress was moderate. The mean scores of the stressors related to clinical learning, academic study, finance and time, confidence, and personal problems ranged from 3.34 to 2.77. The most common coping styles that the nursing students used were support-seeking (mean = 2.84, SD = 0.50), followed by problem-solving (mean = 2.78, SD = 0.42), and venting (mean = 2.77, SD = 0.60). Avoidance coping [e.g., self-blame (mean =2.47, SD = 0.61)], behavioral disengagement (mean = 2.19, SD = 0.66), and substance use (mean = 1.91, SD = 0.85) were used less frequently. The sum score of the C-GHQ-12 ranged from 5 to 30, with a mean = 16.42 (SD = 4.18), indicating that the nursing students experienced psychological distress. There were no significant differences between the genders in overall stress (*p* = 0.978) and psychological distress (*p* = 0.872). Table 1 shows the details of the study variables.

### 4.2. Correlation among Stressors, Coping and Psychological Well-Being

The stressors related to clinical learning, finance and time, confidence, and personal problems—but not academic study—were significantly related to psychological well-being. The nursing students reporting higher overall stress tended to report higher psychological distress (r = 0.257, *p* < 0.001). The nursing students reporting higher psychological well-being tended to use support-seeking, problem-solving, venting, and accommodation to cope with stressors; those who reported lower well-being tended to use self-blame, humor, behavioral disengagement, religion, denial, and substance abuse. For coping with stressors, certain strategies were specifically used. Table 2 shows more details on the associations among the study variables.

### 4.3. Moderating Effects of Coping on Psychological Well-Being

**Figure 1:**
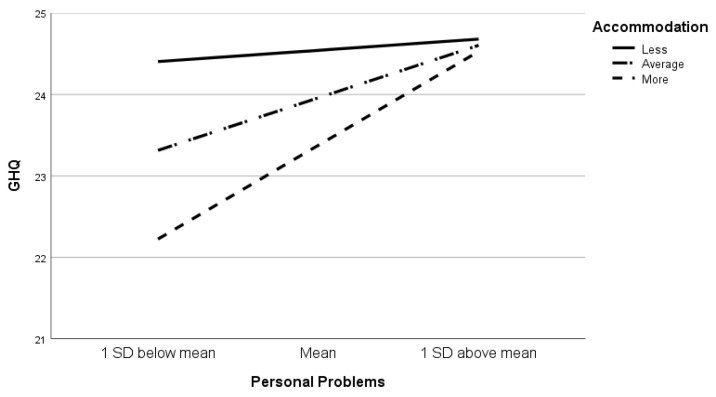
Slope graph testing the interaction between accommodation and personal problems on psychological well-being (GHQ).

Table 3 shows the three models used to test the main effects of stress and coping on psychological well-being. All three models produced statistically significant results. In model 1, the stressors related to clinical learning, confidence, and personal problems predicted the psychological well-being among the nursing students. The stressors related to clinical learning were negatively associated with psychological distress, whereas the stressors related to confidence and personal problems were positively associated with psychological distress. These stressors accounted for 22.6% of the psychological well-being. In model 2, clinical learning and confidence were no longer the predictors of psychological well-being after the use of the coping strategies. The results indicated that problem-solving and accommodation as means of coping protect the psychological well-being. The more these two strategies were used, the less was the distress. In contrast, self-blame and behavioral disengagement were barriers to psychological well-being. The main effects explained 44.5% of the psychological well-being. In model 3, after the use of five interactions between the stressors and the coping strategies, the interaction between the stressors related to personal problems and the coping with accommodation was found to be statistically significant [b = 0.507, *p* = 0.035], indicating that the relationship between personal problems and psychological well-being is moderated by accommodation. The more accommodation was used to cope with personal problems, the higher was the psychological distress. The model explained 45.6% of the variation in the psychological well-being. As shown in Figure 1, there was a direct correlation between the use of accommodation and the GHQ score. It shows that the nursing students who tended to use accommodation less often (the top line) had slightly increased GHQ scores as their personal problems increased. For those who used this strategy more often, i.e., who had an average use of this coping strategy, their GHQ scores showed a moderate increase as their personal problems increased. However, for those who tended to use accommodation as a coping strategy often (the bottom line), the GHQ scores increased greatly as their personal problems increased. This suggests using accommodation as a coping strategy exacerbates the negative effects of personal problems on psychological well-being.

## 5. Discussion

This study aimed to explore the potential sources of stress associated with psychological distress and the moderating effect of coping on the relationship between stress and psychological well-being in final-year Hong Kong nursing students. This is the first study to explore coping strategies not just as predictors of psychological well-being, but also as measures of the extent to which these predictors might have a moderating role between stress and psychological well-being. Results of our study showed that the main sources of stress associated with psychological distress were related to clinical learning, confidence, and personal problems. This finding is consistent with the previous studies that concluded that the lack of knowledge and skills to perform patient care during the clinical placement [7,8,11,12,17], the lack of confidence to deal with interpersonal problems among peers and staff [33,34], and personal problems such as health issues of self and family members [14] might be stressful. Unlike other studies which reported that clinical learning aggravated distress [3,18,35,36], in our study, the nursing students identified clinical learning stress levels as moderate (mean = 3.13; SD = 0.50), while their levels of psychological distress were relatively low. It is possible that stressors related to clinical learning are eustresses [37]. The use of positive coping strategies, such as support-seeking and problem-solving, to cope with the stress in clinical learning might also contribute to relieve the distress.

Our study supports the concepts of Lazarus & Folkmans’ [15] Transactional Model of Stress that claims that coping is an adaptive process based on primary and secondary appraisals. Similar to other studies [8,10,38,39], both problem-focused and emotion-focused coping were used by the nursing students in this study. We found that personal problems and many of the coping strategies (problem-solving, accommodation, self-blame, behavioral disengagement, and substance use) predicted the psychological well-being in the nursing students. As in previous studies, the use of problem-focused coping was associated with lower levels of psychological distress [38]. Accommodation in this study involved both problem-focused coping and emotion-focused coping, characterized by the facets of positive reframing and acceptance, respectively [27]. In this study, accommodation coping was associated with better psychological well-being. Unlike a previous study, which found that emotion-focused coping was associated with depressive symptoms [40], we found that the use of accommodation was adaptive. The apparent difference might be due to the combined facets of the positive reframing and acceptance in accommodation, which represents the ability to yield psychological strength and emotional regulation [27].

Though avoidance coping strategies, such as behavioral disengagement (mean = 2.19, SD = 0.66), denial (mean = 2.03, SD = 0.74), and substance abuse (mean = 1.91, SD = 0.85), were less frequently used here (Table 1), they were strong predictors (Table 2) that have adverse effects on the psychological well-being. These results support the findings of the previous studies that avoidance coping, such as behavioral disengagement, is ineffective [40,41], and is associated with depressive symptoms [42]. Self-blame has been found to be positively associated with negative emotions, such as depression and anxiety [40,43]. Further, the previous studies reported that self-blame is a risk factor for suicidal behavior [44] and a strong predictor of depression in female nursing students [45] The nursing students with low resilience and high burnout tend to use self-blame strategies to cope with stress [46]. Nurse educators must note that strategies should be devised to promote effective coping.

This study adds further evidence that coping strategies moderate stress and psychological well-being. The results of this study showed that using accommodation to cope with the stressors related to personal problems exacerbated the negative effects of the personal problems on psychological well-being. Our study did not collect information about the students’ living conditions, so we might not have sufficient information to assess the social support received by the participants. It should be noted, however, that young people in Hong Kong usually rely on their families financially and live with parents, owing to the high property prices and rental costs. The situation where young people aged 15–24 living are with parents is a rising trend and rose from 91.5% to 94.6% in 2011 [47]. Although young people aged 15–34 reported to have a satisfactory level of family functioning [48], in our study, the stressors related to personal problems included those of both the student and his/her family members, and their relationships with family members. It is possible that final-year nursing students spend more time on their honors project, clinical practice, and preparation for their future career and less time attending to the needs of family members, and even their own health. Hongkongers, as part of a Chinese culture, regard filial piety as a virtue. Conflict with this cultural norm might result in distress if they could not attend to the needs of their parents and family members [49]. Nurse educators and school counsellors should be alerted that final-year nursing students require extra support in handling their personal problems.

It is worth noting that the overall psychological well-being among nursing students in this study was similar to a previous study in which the GHQ scores were higher than the cutoff (>12) [50]. These findings suggest that efforts should be directed to promote effective coping that could improve the psychological well-being of the nursing students.

It should be noted that this study was performed before the outbreak of the COVID-19 pandemic in Hong Kong, so the stressors related to the pandemic could not be assessed. The COVID-19 pandemic indeed disrupted the lives of everyone globally; there is no doubt that the nursing students’ psychological well-being could have been negatively affected [51,52,53,54]. During the COVID-19 pandemic, nursing students reported the sources of stress related to the pandemic included worries about the COVID-19 virus [55], online classes, the financial burden of internet access [56,57], and social restriction [53]. Though the virus is less fatal than it was in 2020, and many countries have entered the endemic stage of the COVID-19 outbreak, the next stage remains uncertain. Thus, nurse educators and counsellors should be sensitive to nursing students’ psychological problems incurred as a result of the pandemic and be able to provide them with appropriate counselling and supportive services.

## 6. Study Limitations

Limitations of this study were mainly related to the cross-sectional design, which does not allow for the establishment of a causal effect. Secondly, we did not collect information about the living conditions might limit the information obtained about the stressors related to personal problems. Thirdly, the respondents were final-year students; a longitudinal study would be needed to monitor the course of stress throughout the nursing program and to assess the sources of stress that contribute to psychological distress. Fourthly, the responses were from self-reported questionnaires; social desirability might compromise the authenticity of the data. Lastly, the results, which were collected before the COVID-19 pandemic, might not reflect the stressors related to the pandemic.

## 7. Conclusions

This study reveals that stressors related to personal problems were associated with psychological distress among nursing students. Problem-solving and accommodation types of coping were adaptive to stress and effective in promoting psychological well-being. Avoidance coping, such as self-blame, was found to be ineffective and had adverse effects on psychological well-being, even when used less frequently. The evidence that accommodation exacerbates the negative effects of personal problems on psychological distress should be noted. Nurse educators should be aware of the different coping styles, should know which are more effective, and should devise educational strategies in the nursing curriculum and create a supportive environment to foster resilience in nursing students to promote those styles.

## Figures and Tables

**Table 1:** Descriptive data of stress, coping style, and psychological well-being, and C-GHQ and C-SINS by gender (N = 293).

				**Mean (SD)**	
1	SINS_Clinical learning			3.13	0.50
2	SINS_Academic study			3.34	0.60
3	SINS_Finance and time			3.01	0.64
4	SINS_Confidence			2.95	0.50
5	SINS_Personal problems			2.77	0.59
6	Stress level_Overall			3.05	0.44
7	COPE_Support seeking			2.84	(0.50)
8	COPE_Problem solving			2.78	(0.42)
9	COPE_Venting			2.77	(0.60)
10	COPE_Accommodation			2.75	(0.43)
11	COPE_Self-distraction			2.73	(0.50)
12	COPE_Self-blame			2.47	(0.61)
13	COPE_Humor			2.31	(0.72)
14	COPE_Behavioral disengagement			2.19	(0.66)
15	COPE_Religion			2.11	(0.82)
16	COPE_Denial			2.03	(0.74)
17	COPE_Substance use			1.91	(0.85)
18	C-GHQ-12			16.42	(4.18)
	**Female**	**Male**	**Total**		
	**n = 233**	**n = 60**	**n = 293**		
	**Mean (SD)**			**Independent *t*-Test ** **(*p* Value)**	
C-GHQ-12	16.45 (3.96)	0.872	16.4 (4.18)	0.872	
SINS-CN	3.05 (0.41)	0.978	3.05 (0.44)	0.978	

SINS: specific subscale of the Stressors in Nursing Students Scale-Chinese version. COPE: specific subscale of the Brief Cope Inventory-Chinese version. C-GHQ-12: Chinese version of the General Health Questionnaire-12. SINS-CN: Stressors in Nursing Students Scale-Chinese version.

**Table 2:** Correlation matrix between stressors, coping and psychological well-being.

	1	2	3	4	5	6	7	8	9	10	11	12	13	14	15	16	17
1																	
2	0.626 ***																
3	0.474 ***	0.399 ***															
4	0.764 ***	0.548 ***	0.575 ***														
5	0.386 ***	0.256 ***	0.502 ***	0.506 ***													
6	0.908 ***	0.691 ***	0.728 ***	0.900 ***	0.594 ***												
7	0.219 ***	0.272 ***	0.121 *	0.117 *	−0.055	0.188 **											
8	0.225 ***	0.203 ***	0.180 **	0.112	0.028	0.205 **	0.418 ***										
9	0.107	0.115 *	0.036	0.061	−0.008	0.088	0.429 ***	0.359 ***									
10	0.189 **	0.123 *	0.020	0.065	−0.023	0.121 *	0.319 ***	0.538 ***	0.249 ***								
11	0.230 ***	0.143 *	0.190 **	0.191 **	0.175 **	0.243 ***	0.282 ***	0.256 ***	0.349 ***	0.324 ***							
12	0.280 ***	0.129 *	0.196 **	0.343 ***	0.302 ***	0.325 ***	0.177 **	0.142 *	0.240 ***	0.089	0.163 **						
13	−0.001	−0.067	0.214 ***	0.159 **	0.219 ***	0.115 *	−0.094	0.140 *	0.029	0.191 **	0.116 *	0.303 ***					
14	−0.068	−0.130 *	0.047	0.102	0.247 ***	0.024	−0.162 **	−0.203 ***	−0.083	−0.179 **	0.056	0.118 *	0.295 ***				
15	−0.192 **	−0.238 ***	−0.087	−0.054	0.075	−0.143 *	−0.021	−0.062	−0.097	−0.063	−0.006	0.117 *	0.297 ***	0.317 ***			
16	−0.132 *	−0.287 ***	0.087	0.033	0.248 ***	−0.035	−0.170 **	−0.177 **	−0.098	−0.143 *	0.005	0.119 *	0.398 ***	0.530 ***	0.336 ***		
17	−0.251 ***	−0.365 ***	−0.025	−0.029	0.192 **	−0.149 *	−0.266 ***	−0.221 ***	−0.136 *	−0.183 **	−0.026	0.153 **	0.379 ***	0.512 ***	0.419 ***	0.619 ***	
18	0.138 *	0.041	0.216 ***	0.313 ***	0.391 ***	0.257 ***	−0.167 **	−0.235 ***	−0.062	−0.266 ***	−0.001	0.391 ***	0.250 ***	0.414 ***	0.179 **	0.313 ***	0.364 ***

* p < 0.05; ** *p* < 0.01; *** *p* < 0.001. Number 1–6 refer to the specific subscale of the Stressor in Nursing Students Scale-Chinese version. Number 7–17 refer to the specific subscale of the Brief Cope Inventory-Chinese version. Number 18 refers to the Chinese version of the General Health Questionnaire-12.

**Table 3:** Predictors of stressors and coping on psychological well-being using multiple hierarchical regression.

	Model 1	Model 2	Model 3
	β	β	β
Gender	0.410	−0.009	0.031
Age	−0.332	−0.299	−0.330
Institution	−0.107	−0.131 *	−0.131 *
Religious belief	1.036	0.205	0.179
SINS_Clinical learning	−0.752 *	−0.005	0.012
SINS_Academic study	−0.479	0.121	0.103
SINS_Finance and time	−0.127	0.081	0.072
SINS_Confidence	1.524 ***	0.518	0.497
SINS_Personal problems	1.413 ***	0.640 *	0.645 *
COPE_Support seeking		−0.234	−0.218
COPE_Problem_solving		−0.584 *	−0.621 *
COPE_Venting		0.035	0.017
COPE_Accommodation		−0.620 *	−0.583 *
COPE_Self distraction		−0.088	−0.096
COPE_Self blame		1.138 ***	1.133 ***
COPE_Humor		0.233	0.198
COPE_Behavioral disengagement		0.863 **	0.923 ***
COPE_Religion		−0.006	−0.004
COPE_Denial		−0.044	−0.026
COPE_Substance use		0.541	0.582 *
SIN_Personal problems x COPE_Problem solving			−0.433
SIN_Personal problems x COPE_Accommodation			0.507 *
SIN_Personal problems x COPE_Self blame			0.216
SIN_Personal problems x COPE_Behavioral disengagement			−0.041
SIN_Confidence x COPE_Behavioral disengagement			0.102
**R^2^**	0.226	0.445	0.456
**Adjusted R^2^**	0.202	0.404	0.405
**R^2^ change**	0.226	0.219	0.456
**F**	9.206 ***	10.917 ***	8.959 ***

Model 1: df1 = 9, df2 = 283; Model 2: df1 = 11, df2 = 272; Model 3: df1 = 25, df2 = 267. β = unstandardized regression estimates. Religious belief: 1 = No, 2 = Yes. Dummy variables for Institution A = 0, 1, institution B = 1, 0 = institution C = 0, 0. Gender: 1 = Male; 2 = Female. * *p* < 0.05; ** *p* ≤ 0.01; *** *p* ≤ 0.001.

## Data Availability

Not applicable.
